# Pan-cancer analysis identifies NT5E as a novel prognostic biomarker on cancer-associated fibroblasts associated with unique tumor microenvironment

**DOI:** 10.3389/fphar.2022.1064032

**Published:** 2022-12-07

**Authors:** Xin-miao Xue, Yu-yang Liu, Xue-min Chen, Bing-yan Tao, Peng Liu, Han-wen Zhou, Chi Zhang, Li Wang, Yu-ke Jiang, Zhi-wei Ding, Wei-dong Shen, Jun Zhang, Shi-ming Yang, Fang-yuan Wang

**Affiliations:** ^1^ Medical School of Chinese People’s Liberation Army (PLA), Beijing, China; ^2^ Senior Department of Otolaryngology-Head & Neck Surgery, Chinese People’s Liberation Army (PLA) General Hospital, National Clinical Research Center for Otolaryngologic Diseases, State Key Lab of Hearing Science, Beijing Key Lab of Hearing Impairment Prevention and Treatment, Ministry of Education, Beijing, China; ^3^ Department of Neurosurgery, Chinese People’s Liberation Army (PLA) General Hospital, Beijing, China; ^4^ The Zhantansi Outpatient Department of Central Medical Branch of People’s Liberation Army (PLA) General Hospital Beijing, China

**Keywords:** NT5E, CD73, pan-cancer analysis, cancer-associated fibroblast, immunotherapy, epithelial-mesenchymal transition

## Abstract

**Background:** Ecto-5′-nucleotidase (NT5E) encodes the cluster of differentiation 73 (CD73), whose overexpression contributes to the formation of immunosuppressive tumor microenvironment and is related to exacerbated prognosis, increased risk of metastasis and resistance to immunotherapy of various tumors. However, the prognostic significance of NT5E in pan-cancer is obscure so far.

**Methods:** We explored the expression level of NT5E in cancers and adjacent tissues and revealed the relationship between the NT5E expression level and clinical outcomes in pan-cancer by utilizing the UCSC Xena database. Then, correlation analyses were performed to evaluate the relationship between NT5E expression and immune infiltration level *via* EPIC, MCP-counter and CIBERSORT methods, and the enrichment analysis were employed to identify NT5E-interacting molecules and functional pathways. Furthermore, we conducted single-cell analysis to explore the potential role of NT5E on single-cell level based on the CancerSEA database. Meanwhile, gene set enrichment analysis (GSEA) in single-cell level was also conducted in TISCH database and single-cell signature explorer was utilized to evaluate the epithelial-mesenchymal transition (EMT) level in each cell type.

**Results:** The expression level of NT5E was aberrant in almost all cancer types, and was correlated with worse prognosis in several cancers. Notably, NT5E overexpression was related to worse overall survival (OS) in pancreatic adenocarcinoma (PAAD), head and neck squamous cell carcinoma (HNSC), mesothelioma (MESO), stomach adenocarcinoma (STAD), uveal melanoma (UVM) and cervical squamous cell carcinoma and endocervical adenocarcinoma (CESC) (*p* < 0.01). NT5E-related immune microenvironment analysis revealed that NT5E is associated positively with the degree of infiltration of cancer-associated fibroblasts (CAFs) and endothelial cells in most cancers. Enrichment analysis of cellular component (CC) demonstrated the critical part of NT5E played in cell-substrate junction, cell-substrate adherens junction, focal adhesion and external side of plasma membrane. Finally, single-cell analysis of NT5E illuminated that EMT function of CAFs was elevated in basal cell carcinoma (BCC), skin cutaneous melanoma (SKCM), HNSC and PAAD.

**Conclusion:** NT5E could serve as a potential prognostic biomarker for cancers. The potential mechanism may be related to the upregulated EMT function of CAFs, which provides novel inspiration for immunotherapy by targeting CAFs with high NT5E expression.

## Highlights

1) NT5E could serve as an efficient prognostic biomarker in pan-cancer.
2) NT5E expression is positively related to cancer-associated fibroblasts (CAFs) and endothelial cells infiltration in pan-cancer.
3) NT5E is highly expressed in the endothelia cells and CAFs in pan-cancer.
4) CAFs may play an important role in epithelial-mesenchymal transition (EMT) of various tumor species, which may be a novel target for immunotherapy.

## 1 Introduction

Ecto-5′-nucleotidase (NT5E), namely cluster of differentiation 73 (CD73), is a glycosylphosphatidylinositol-anchored cell surface protein. Encoded by the NT5E gene, CD73 is widely distributed in the human body, including the central nervous system, cardiovascular system, and epithelial tissues (Thompson et al., 2004; Zimmermann et al., 2012; Jeong et al., 2020). Structurally, NT5E consists of three domains, including a glycosylated N-terminal domain and a C-terminal domain, which are responsible for metal binding and the catalytic function respectively, and an alpha helix connecting the aforementioned two domains (Buschette-Brambrink and Gutensohn, 1989; Fini et al., 2003). Functionally, NT5E possesses nucleosidase activity (Sträter, 2006), and could hydrolyze extracellular adenosine monophosphate (AMP) into adenosine (Kordas et al., 2018). Extracellular adenosine plays an important role in modulating inflammation regulation and tumor immunity (Antonioli et al., 2013; Allard et al., 2017a; Kordas et al., 2018; Boison and Yegutkin, 2019), where A_2A_ receptor (A_2A_R)-mediated signaling pathway matters most (Colella et al., 2018). Adenosine can activate the immune suppressive effects, which is characterized by the inhibition of chemotaxis and proliferation function among T cells (Sitkovsky et al., 2004; Allard et al., 2014). Meanwhile, it has been demonstrated that adenosine promotes angiogenesis and inhibits the release of cytokines and the expression of adhesion molecules such as E-selectin (Bouma et al., 1996; Spychala, 2000), hinting that NT5E may be related to cell adhesion function (Henttinen et al., 2003). Furthermore, it has also been proved that NT5E could influence cell adhesion and migration performance by the molecular mechanism of tenascin C, one of the important factors among extracellular matrix (ECM) (Sadej and Skladanowski, 2012). Therefore, NT5E could promote tumor growth not only by accumulating adenosine to inhibit the antitumoral immune responses, but also by facilitating dissemination of cancer cells (Kordas et al., 2018).

According to previous studies, NT5E has been detected among various tumor entities, including melanoma (Sadej et al., 2006a; Sadej et al., 2006b; Wang et al., 2012), triple-negative breast cancer (Allard et al., 2014; Buisseret et al., 2018), colorectal cancer (Liu et al., 2012), and non-small cell lung cancer (Inoue et al., 2017). Furthermore, it is not only expressed on malignant cells, but also on several immune cells such as regulatory T cells (Tregs) (Alam et al., 2009), myeloid-derived suppressor cells (MDSCs) (Ryzhov et al., 2011), dendritic cells (DCs) (Berchtold et al., 1999) and natural killer (NK) cells (Neo et al., 2020), which could result in more obvious accumulation of immunosuppressive adenosine and lead to the downregulation of the T cell immune responses (Saldanha-Araujo et al., 2011), and it has been illustrated that NT5E^+^ NK cells could inhibit T cell activity by upregulating interleukin-10 (IL-10) and transforming growth factor-β (TGF-β) production (Neo et al., 2020). Moreover, adenosinergic A_2A_R were also expressed on DCs, MDSCs, NK cells, and macrophages, indicating that the function of these regulatory immune cells could also be inhibited by adenosine (Allard et al., 2016; Kalekar et al., 2016; Young et al., 2016; Kalekar and Mueller, 2017).

It has been illustrated that adenosine triphosphate (ATP) concentration is about hundreds of thousands of times higher in the tumor microenvironment (TME) than the non-tumoral extracellular tissues (Zimmermann, 2000; Pellegatti et al., 2008). ATP is hydrolyzed to AMP, and lastly to adenosine by plasma membrane nucleotidases. According to previous studies, adenosine-mediated immunosuppression is a crucial part in the TME (Leone and Emens, 2018), which was constructed by vascular endothelial cells, fibroblast cells, and many types of innate and adaptive immune cells, together with ECM as well as multiple extracellular soluble molecules (cytokines, chemotactic factor, growth factors, etc.) (Binnewies et al., 2018). Furthermore, TME complexity is an important part in the differentiation of cold tumors and hot tumors. The feature of hot tumors is a high T cell infiltration level and abundant immune active molecular signatures, while cold tumors show distinctive characteristic of T cell absence (Gajewski, 2015). Thus, the immunosuppressive environment of cold tumors exhibited resistant to numerous immune checkpoint blockade therapies (Quail and Joyce, 2017).

There are several components correlated with the maintenance of an immunosuppressive environment, including some molecules like TGF-β, epidermal growth factor (EGF) and adenosine (Wei et al., 2022), and several immunosuppressive cells, including Tregs, tumor-associated macrophages (TAMs), endothelial cells, and cancer-associated fibroblasts (CAFs) (Zhu et al., 2022). In particular, CAFs may correlate with the enhancement of tumor phenotypes by regulating cancer cell proliferation and invasion and ECM remodeling (Costa et al., 2014; Gascard and Tlsty, 2016; Gentric et al., 2017). It has been validated that CAFs could secrete a vast amount of cytokines, including hepatocyte growth factor, EGF, connective tissue growth factor, insulin‐like growth factor. All these cytokines could function directly on the surrounding cells and facilitate ECM reprogramming. Meanwhile, CAFs also secrete extracellular vesicles, metabolites, ECM components and ECM‐remodeling enzymes (Jacob et al., 2012). Consequently, CAFs were considered to play a long-term role in the tumor development from tumorigenesis to cancer metastasis (Cirri and Chiarugi, 2011; Marsh et al., 2013; Kalluri, 2016).

In our previous study, we have concluded that NT5E could serve as an independent prognostic indicator for head and neck squamous cell carcinoma (HNSC) (Chen et al., 2022). Similarly, in pancreas, prostate and bladder cancer, NT5E has also been validated to correlate with tumor development and invasion (Yang et al., 2013; Mandapathil et al., 2018; Koivisto et al., 2019; Zhou et al., 2019; Chen et al., 2022). In detail, for gastric cancer patients, CD73 may serve as a regulator in RICS/RhoA-LIMK-cofilin signaling pathway by its extracellular function in adenosinergic pathway, and then promote β-catenin-induced epithelial-mesenchymal transition (EMT) process, which is correlated with metastasis property of tumor cells (Xu et al., 2020; Goulioumis and Gyftopoulos, 2022). In our study, the enrichment analyses also indicated that NT5E may be related to EMT and metastasis during HNSC progression. Furthermore, HNSC-related immune infiltration analysis and single-cell type analysis revealed that NT5E expression was positively related to CAFs infiltration in HNSC (Chen et al., 2022), which is in line with the previous conclusion that NT5E expression is related to tumor migration and invasion (Costa et al., 2014).

Although there is abundant evidence indicating that the expression level of NT5E is related to clinical outcomes and the prognosis in certain tumors, several questions still remain suspension, including the expression landscape of NT5E among various tumor types, the certain cell types expressing NT5E, and the potential signal pathways consisting NT5E in tumor growth and metastasis. To the best of our knowledge, the pan-cancer analysis of NT5E is still a virgin land. Thus, in this study, we performed NT5E expression analysis and prognosis analysis in pan-cancer, and explored the potential role of NT5E in the TME and the EMT function of CAFs, so as to provide novel clues for immunotherapy against malignant tumors.

## 2 Materials and methods

### 2.1 Dataset acquisition and normalization

The data of mRNA expression profile and clinical outcomes of patients (TCGA pan-cancer cohort) or normal tissues (GTEx database) were acquired from the UCSC Xena database (https://xenabrowser.net/datapages/). By applying the transcripts per million (TPM) method, we normalized the raw data. Then we employed log2 (TPM+1) transformation for the subsequent analyses. The information of genomic alteration frequency about NT5E in the 33 cancer types were acquired from the cBioPortal database (http://cbioportal.org).

### 2.2 NT5E expression analysis

Based on the mRNA expression profile obtained from UCSC Xena database, the NT5E expression level was compared between tumors and corresponding normal tissues. Totally, 31 types of tumors were included in this analysis, except for mesothelioma (MESO) and uveal melanoma (UVM), because of unavailability of corresponding normal tissues data. Besides, NT5E expression in patients stratified by different characteristics were also compared. The R software (Version 3.6.3) was used for statistical analysis with “ggplot2” package adopted for visualization. Moreover, the representative NT5E immunohistochemistry (IHC) staining pictures were retrieved from the Human Protein Atlas (HPA) on line database (http://www.proteinatlas.org).

### 2.3 Single-cell analysis of NT5E

To uncover the potential role of NT5E on single-cell level, we used the CancerSEA database (http://biocc.hrbmu.edu.cn/CancerSEA/home.jsp) to reveal the relationship between NT5E expression level and 14 function status in distinct cancers. Moreover, the Tumor Immune Single-cell Hub (TISCH) database (http://tisch.comp-genomics.org/home/) were employed to quantify the expression level of NT5E in different cell type. Gene set enrichment analysis (GSEA) in single-cell level was also conducted in TISCH database. Up-regulated hallmark gene-sets were visualized in the heatmap. Meanwhile, we used single-cell signature explorer to evaluate the level epithelial mesenchymal transition in each cell type.

### 2.4 Prognostic value of NT5E in pan-cancer

The prognosis information including overall survival (OS), disease-specific survival (DSS), disease-free interval (DFI) and progression-free interval (PFI) was obtained from the UCSC Xena database (https://xenabrowser.net/datapages/). The continuous variable of NT5E expression profile was utilized in the univariate Cox regression analysis. Meanwhile, the Kaplan–Meier curve was also used to evaluate the prognostic value of NT5E, and the cut-off point with the minimum *p*-value was selected for further analysis. The “survival” package was applied for statistical analysis, and the “survminer” package was used for visualization.

### 2.5 NT5E-related immune microenvironment analysis

For NT5E-related immune infiltration analysis, three methods (EPIC, MCP-counter, and CIBERSORT) were selected for further analysis. Correlation analyses were utilized to estimate the relationship between NT5E expression and immune infiltration level. All these immune infiltration levels of each sample were directly acquired from the TIMER2.0 database (http://timer.comp-genomics.org/). The heatmap constructed by the “ggplot2” R package was used for results visualization.

### 2.6 Identification of NT5E related molecules and functional enrichment

The top 50 NT5E-associated proteins were obtained *via* STRING database (https://cn.string-db.org/). Briefly, the parameters were selected as follows: evidence is selected for meaning network edges, all options were included for active interaction sources, and the medium confidence was chosen at 0.4 for minimum required interaction score. The Cytoscape software (Version 3.9.1) was utilized for visualization. Additionally, an NT5E-related gene-gene interaction (GGI) network was constructed using the GeneMANIA database (http://www.genemania.org), and the top 20 genes most closely to NT5E were involved in GGI. Besides, the top 100 co-expressed genes of NT5E were obtained from the COXPRESdb (Obayashi et al., 2019) (https://coxpresdb.jp/). The upset diagram was used to illustrate NT5E-related molecules from these three online databases and “UpSetR” R package was utilized for visualization. Totally, 168 NT5E related molecules were selected to perform enrichment analysis using the R package “clusterProfiler” and the “ggplot2” package was used for visualization.

### 2.7 Immunofluorescence staining

We collected supraglottic carcinoma specimens from the operating room of Chinese PLA General Hospital. All specimens were fixed with 4% formalin and embedded in paraffin. Seven serial sections with a thickness of 3 mm were made. After using high pressure method for antigen retrieval for 3 min, the sections were blocked with 10% goat serum (C0265, Beyotime, China) for 30 min in thermostat at 37°C, and incubated overnight at 4°C with smooth muscle actin (α-SMA/ACTA2) primary antibody at concentrations of 1:50 (CL594-14395, Proteintech, United States). Then, sections were rinsed with PBS for three times for 10 min each, and incubated with NT5E antibody at 1:50 (CL488-67789, Proteintech, United States) overnight at 4°C. The cy3-labeled goat anti-rabbit IgG (A0516, Beyotime, China) was added and incubated at room temperature for 1 h, followed by counterstaining with DAPI for 8 min. Whole slide imaging was operated by Pannoramic scan system (3DHISTECH, Hungary). The abovementioned procedures were approved by the Ethics Committee of Chinese PLA General Hospital (No. S2021-339-02). All of the patients or their legal guardians gave their informed consent to participate.

### 2.8 Statistical analysis

The Wilcoxon rank-sum test was employed to detect the statistical significance between two groups. Correlation analysis was analyzed by Spearman’s correlation coefficient. All statistical analysis was performed using R software (version 3.6.3), and two-tailed *p* < 0.05 was considered as of statistical significance.

## 3 Results

### 3.1 NT5E was aberrantly expressed in pan-cancer

**FIGURE 1:**
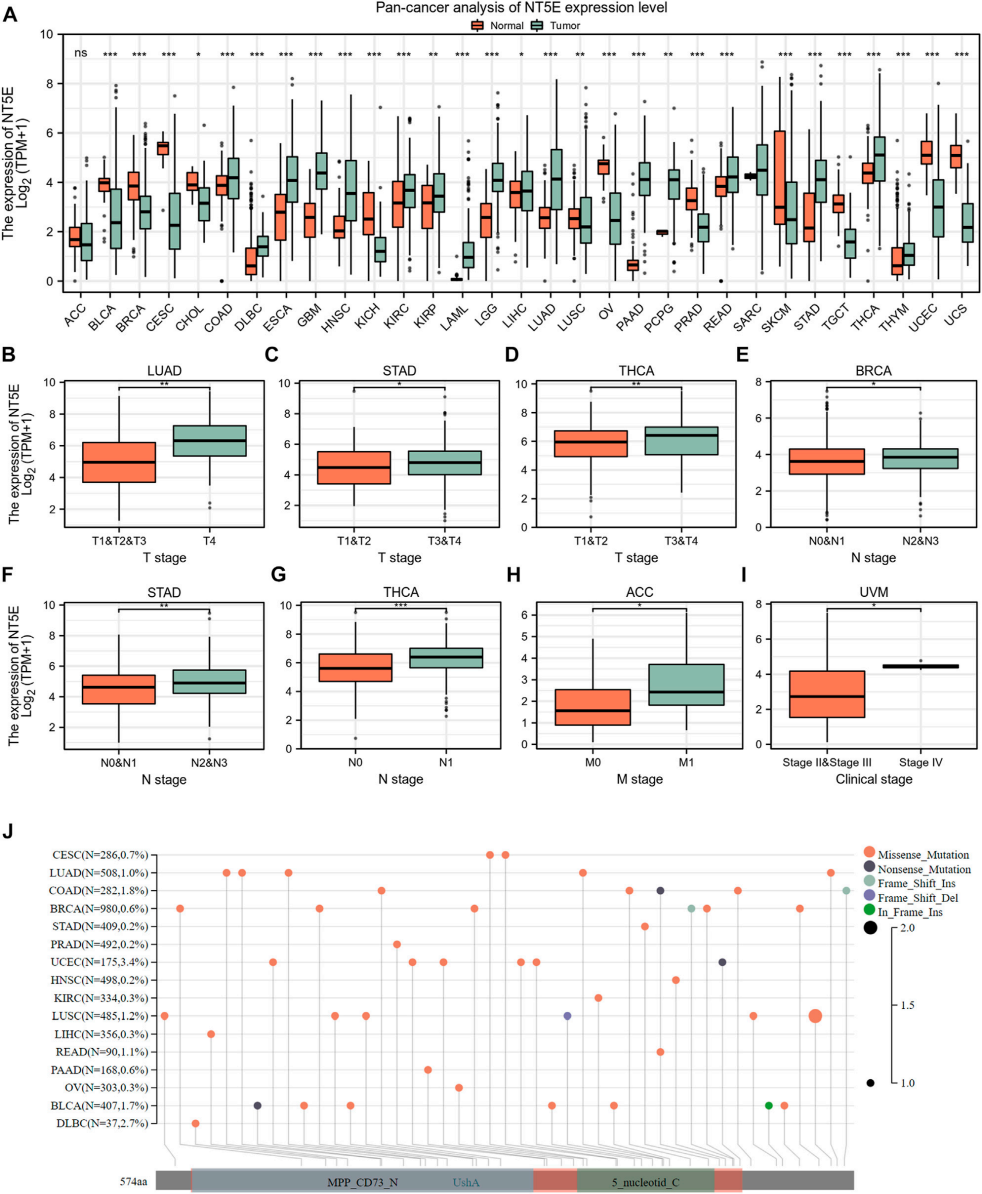
Basic information of NT5E. **(A)** The expression level of NT5E between tumor and normal tissues in each cancer based on the integrated data from TCGA and GTEx databases. **(B)** The expression level of NT5E between T1 & T2 & T3 and T4 stage in LUAD. **(C)** The expression level of NT5E between T1 & T2 and T3 & T4 stage in STAD. **(D)** The expression level of NT5E between T1 & T2 and T3 & T4 stage in THCA. **(E)** The expression level of NT5E between N0 & N1 and N2 & N3 stage in BRCA. **(F)** The expression level of NT5E between N0 & N1 and N2 & N3 stage in STAD. **(G)** The expression level of NT5E between N0 and N1 stage in THCA. **(H)** The expression level of NT5E between M0 and M1 stage in ACC. **(I)** The expression level of NT5E between clinical stage II & stage III and clinical stage IV in UVM. **(J)** NT5E abrupt landscape map in pan-cancer study according to the cBioPortal database (ns, *p* ≥ 0.05, *p < 0.05, **p < 0.01, ***p < 0.001).

To illuminate the expression landscape of NT5E in cancer, we performed studies comparing NT5E mRNA expression level in cancers and normal tissues *via* TCGA and GTEx databases. The results indicated that the expression level of NT5E is significantly aberrant in a variety of cancer types. It was up-regulated in tumoral tissues compared to normal tissues in colon adenocarcinoma (COAD), lymphoid neoplasm diffuse large B-cell lymphoma (DLBC), esophageal carcinoma (ESCA), glioblastoma multiforme (GBM), HNSC, kidney renal clear cell carcinoma (KIRC), kidney renal papillary cell carcinoma (KIRP), acute myeloid leukemia (LAML), brain lower grade glioma (LGG), lung adenocarcinoma (LUAD), pancreatic adenocarcinoma (PAAD), rectum adenocarcinoma (READ), stomach adenocarcinoma (STAD), thyroid carcinoma (THCA) and thymoma (THYM) (*p* < 0.001) (Figure 1A), while down-regulated in bladder Urothelial Carcinoma (BLCA), breast invasive carcinoma (BRCA), cervical squamous cell carcinoma and endocervical adenocarcinoma (CESC), kidney chromophobe (KICH), ovarian serous cystadenocarcinoma (OV), prostate adenocarcinoma (PRAD), skin cutaneous melanoma (SKCM), testicular germ cell tumors (TGCT), uterine corpus endometrial carcinoma (UCEC) and uterine carcinosarcoma (UCS) (*p* < 0.001) (Figure 1A).

Furthermore, in order to illustrate the relationship between NT5E overexpression and tumor progression, we employed studies to analyze the degree of NT5E expression in different pathological stages and revealed an aberrant difference in NT5E expression as the tumor progressed in adrenocortical carcinoma (ACC), uveal melanoma (UVM), LUAD, STAD, BRCA, and THCA. Firstly, NT5E expression is linked to cancer T stage, which was elevated in T4 stage compared with T1 & T2 & T3 stage in LUAD (*p* < 0.01) (Figure 1B), and was higher in T3 & T4 stage than T1 & T2 stage in STAD (*p* < 0.05) and THCA (*p* < 0.01) (Figures 1C,D). Secondly, expression levels of NT5E were also corelated with cancer N stage. We illustrated that the expression level of NT5E up-regulated in N2 & N3 stage in BRCA (*p* < 0.05) and STAD (*p* < 0.01) **(** Figures 1E,F **)**, and up-regulated in N1 stage compared with N0 stage in THCA (*p* < 0.001) (Figure 1G). Finally, the expression level of NT5E is also different between M0 and M1 stage in ACC (*p* < 0.05) (Figure 1H), and difference between clinical stage II & III and clinical stage IV in UVM (*p* < 0.05) (Figure 1I). And then, we performed genomic alteration analysis of NT5E and the results illuminated that alterations of NT5E across pan-cancer were not universal (Figure 1J). Thus, we hypothesized that it may be more important to study the changes of NT5E expression in transcription levels.

### 3.2 NT5E could serve as an efficient prognostic biomarker in pan-cancer

**FIGURE 2:**
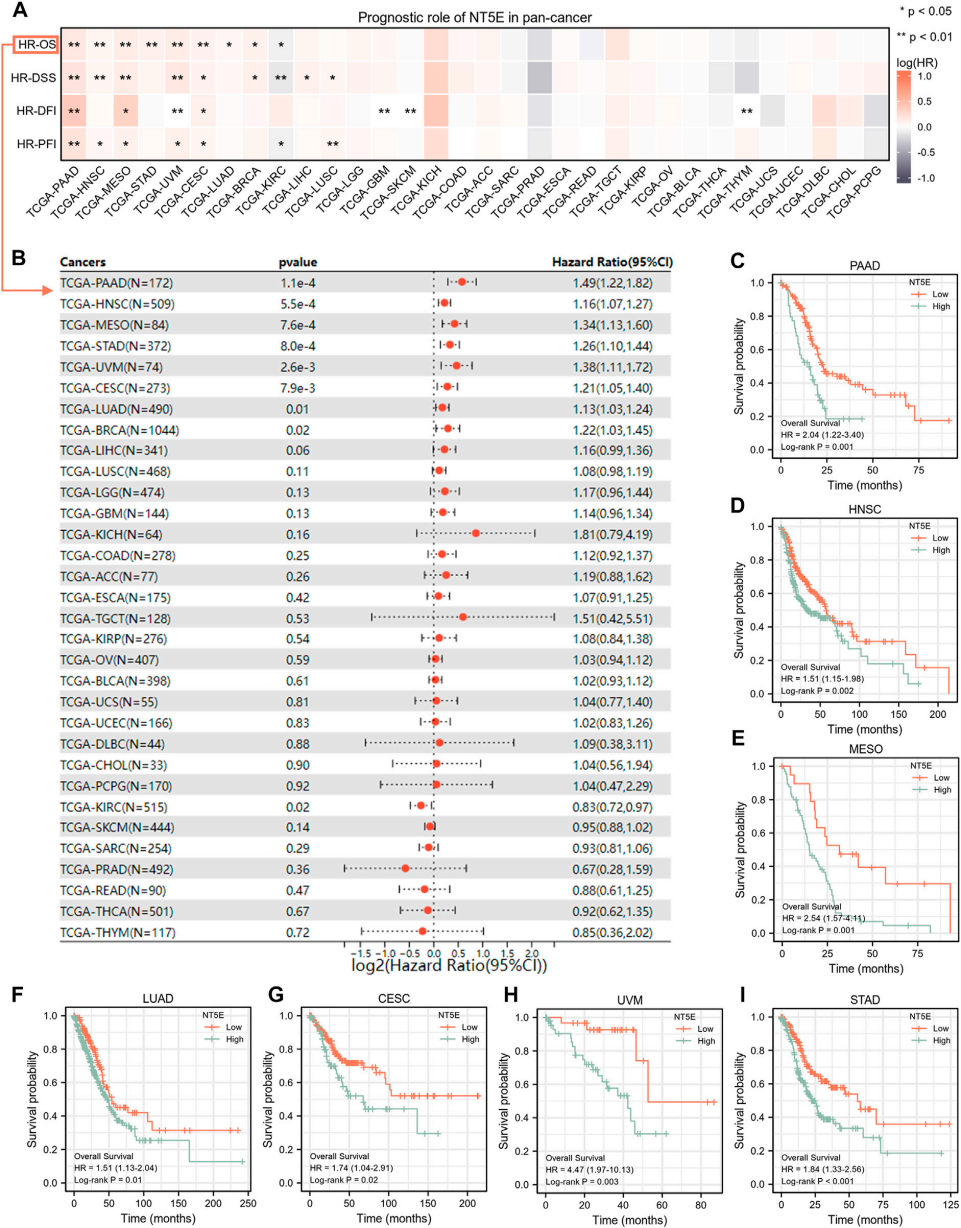
**(A)** the relationship between expression level of NT5E and overall survival (OS), disease-specific survival (DSS), disease-free interval (DFI) and progression-free interval (PFI) through the univariate Cox regression and Kaplan-Meier models. Red represents that NT5E is a risk factor, and gray indicates a protective factor related to prognosis. Only *p* values < 0.05 were shown. **(B)** The forest plot showed the prognostic value of NT5E in cancers using univariate Cox regression method. **(C–I)** Kaplan-Meier overall survival curves of NT5E in PAAD (HR = 2.04; 95% CI = 1.22–3.40; *p* = 0.001) **(C)**, HNSC (HR = 1.51; CI = 1.51–1.98; *p* = 0.002) **(D)**, MESO (HR = 2.54; CI = 1.57–4.11; *p* = 0.001) **(E)**, LUAD (HR = 1.51; CI = 1.13–2.04; *p* = 0.01) **(F)**, CESC (HR = 1.74; CI = 1.04–2.91; *p* = 0.02) **(G)**, UVM (HR = 4.47; CI = 1.97–10.13; *p* = 0.003) **(H)**, and STAD (HR = 1.84; CI= 1.32–2.56; *p* < 0.001) **(I)** (*p < 0.05; **p < 0.01).

We performed studies to validate the potential value of NT5E expression in clinical prognostic among pan-cancer patients derived from TCGA database. According to the univariate Cox regression analysis, NT5E expression was associated with a variety of prognostic indicators in a variety of tumors, and the overexpression of NT5E could strongly predict worse OS in pancreatic adenocarcinoma (PAAD), HNSC, mesothelioma (MESO), STAD, UVM, CESC (*p* < 0.01), LUAD, BRCA and KIRC (*p* < 0.05) (Figures 2A,B). Confounding characteristics selected with *p* < 0.05, we then conducted Kaplan-Meier survival analysis, which suggested that a higher NT5E expression was associated with poor survival outcomes in PAAD [hazard ratio (HR) = 2.04; 95% confidence interval (CI) = 1.22–3.40; *p* = 0.001), HNSC (HR = 1.51; CI = 1.51–1.98; *p* = 0.002), MESO (HR = 2.54; CI = 1.57–4.11; *p* = 0.001), LUAD (HR = 1.51; CI = 1.13–2.04; *p* = 0.01), CESC (HR = 1.74; CI = 1.04–2.91; *p* = 0.02), UVM (HR = 4.47; CI = 1.97–10.13; *p* = 0.003), and STAD (HR = 1.84; CI = 1.32–2.56; *p* < 0.001), emphasizing that overexpression of NT5E is related to poor prognosis in these cancers (Figures 2C–I).

### 3.3 NT5E expression was positively related to CAFs and endothelial cells infiltration in pan-cancer

**FIGURE 3:**
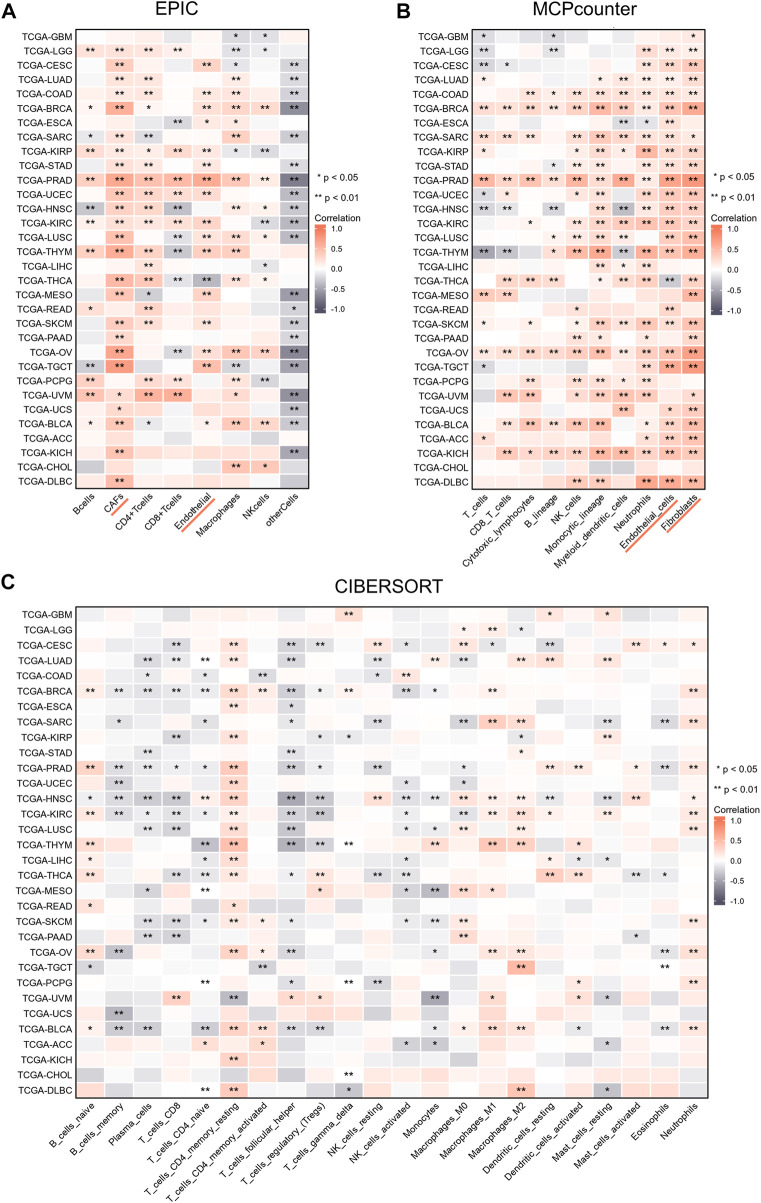
**(A–C)** The relationships of NT5E expression and the infiltration levels of immune cells in cancers based on EPIC **(A)**, MCPcounter **(B)**, and CIBERSORT methods **(C)**. Positive correlation in red and negative correlation in gray. (*p < 0.05; **p < 0.01).

We performed studies to analyze the relationship between the infiltration degree of the immune cells and NT5E expression by several algorithms. According to EPIC and MCPcounter algorithms, we observed that NT5E expression was positively related to CAFs and endothelial cells infiltration in almost all cancers (Figures 3A,B; *p* < 0.01). Furthermore, to reveal the relationship between more species immune cells infiltration situation and the expression level of NT5E, we employed related analysis by applying CIBERSORT algorithms, in which there are 22 kinds of cells. The results illustrated that the expression level of NT5E is positively correlated several immune cells infiltration in almost all cancers, such as memory CD4^+^ T-Cells and M1-polarized macrophages, while negatively related to plasma cells and T follicular helper cells in pan-cancer (Figure 3C).

### 3.4 NT5E was related to cell adhesion function

**FIGURE 4:**
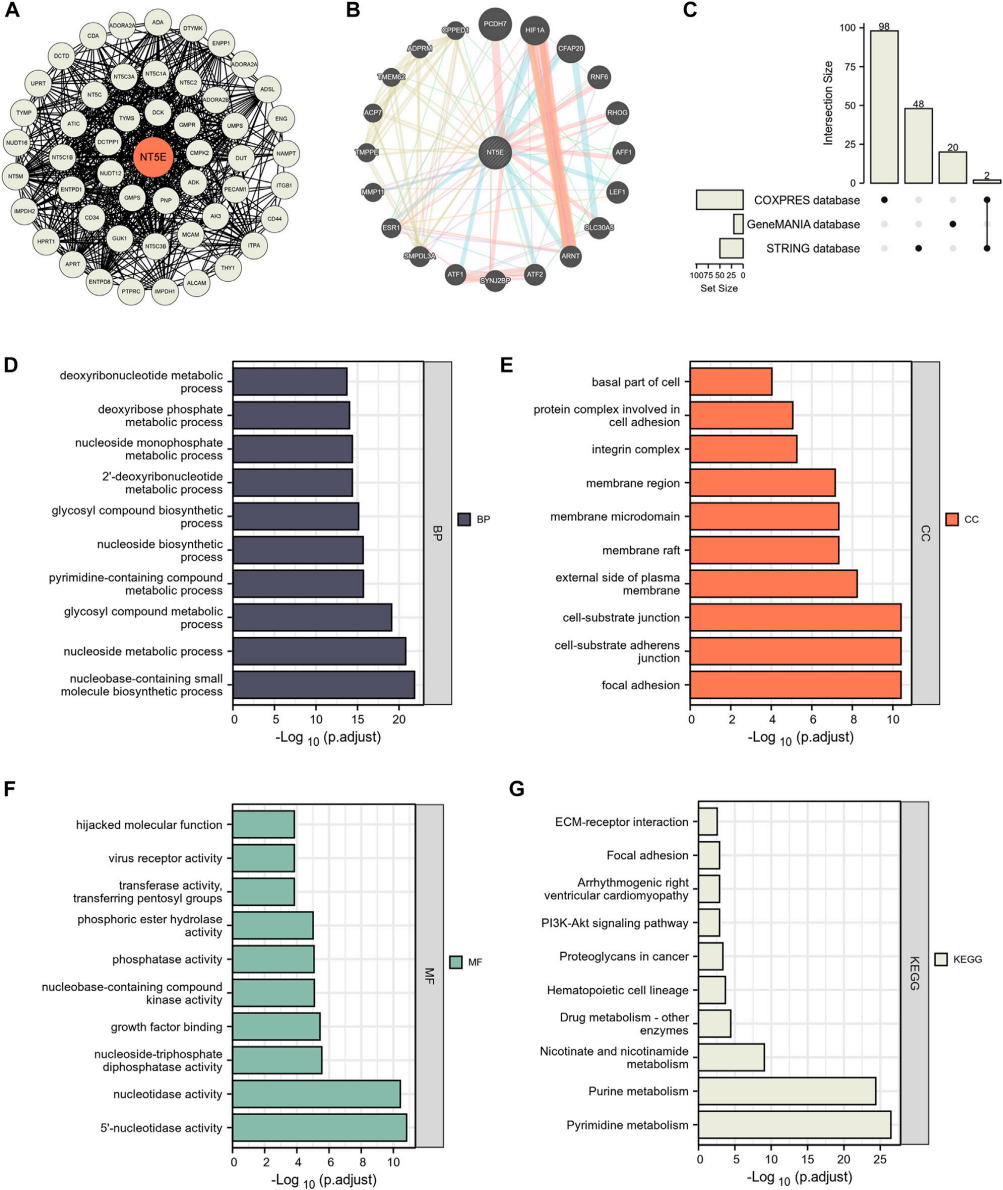
NT5E-related gene enrichment analysis. **(A)** We obtained the available experimentally determined NT5E -binding proteins using the STRING database. **(B)** We used the GeneMANIA website to get the 20 genes most closely related to NT5E. **(C)** 168 NT5E related molecules were selected from STRING database, GeneMANIA database and COXPRESdb. **(D–F)** The biological process **(D)**, cell components **(E)**, and molecular function **(F)** involved in NT5E in GO enrichment analyses. **(G)** the KEGG pathways enrichment analysis.

We have confirmed that the expression degree of NT5E is corelated with the immune cell infiltration in the TME of several cancers. Thus, it is necessary to further explore the potential function of NT5E in the TME. We obtained numerous proteins that closely contacted with NT5E in functional level through the STRING database, in which the data was verified by experimental evidence. The protein interaction network was exhibited in Figure 4A, and the gene interaction network was exhibited in Figure 4B, which was analysed based on the GeneMANIA website. Furthermore, we performed GO and KEGG enrichment analysis based on 168 NT5E related molecules, which were selected from STRING database, GeneMANIA database and COXPRESdb (Figure 4C). The GO enrichment analysis revealed the biological process (BP), cellular component (CC) and molecular function (MF), involved in NT5E (Figures 4D–F). Moreover, the result of KEGG enrichment analysis is exhibited in Figure 4G. It’s worth noting that the cell components related to NT5E include cytoplasmic vesicle lumen, cell-substrate junction, cell-substrate adherens junction, focal adhesion and external side of plasma membrane (Figure 4E).

### 3.5 NT5E was highly expressed in the endothelia cells and CAFs in pan-cancer

**FIGURE 5:**
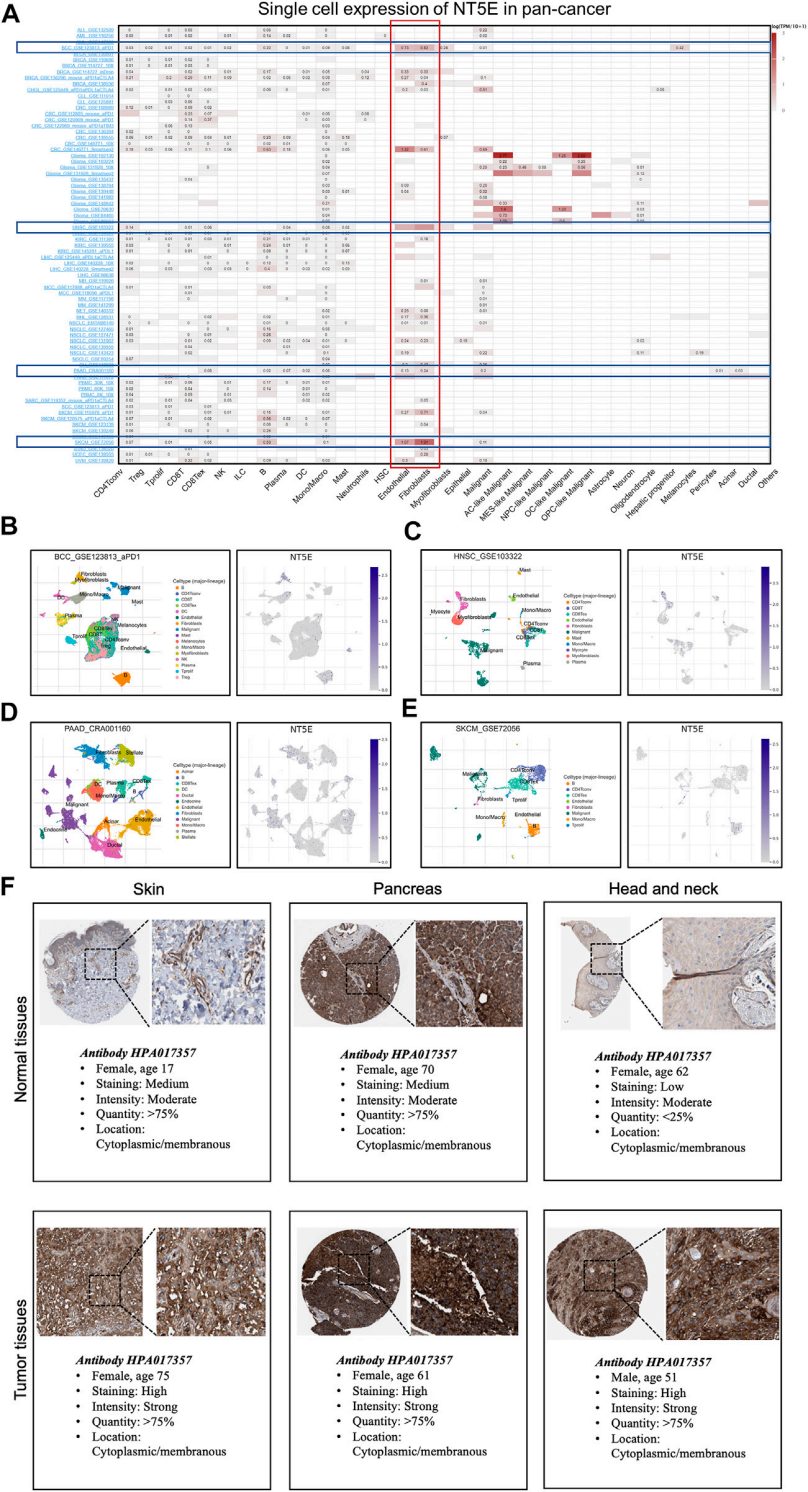
**(A)** Summary of NT5E expression of 33 cell types in 79 single cell databases. **(B–E)** Scatter plot showed the distributions of 10 different cell types (Left) and the NT5E expression levels (Right) of cells in the GSE123813_aPD1 BCC database **(B)**, GSE103322 HNSC database **(C)**, CRA001160 PAAD database **(D)** and GSE72056 SKCM database **(E)**. **(F)** Expression of the NT5E protein in several normal and tumor tissues.

We performed the single-cell analysis of NT5E to quantify the expression level of NT5E in different cell types (including immune cells, stromal cells, malignant cells, and functional cells), the results also showed that NT5E more likely expressed in the endothelia cells and CAFs in several cancers such as basal cell carcinoma (BCC), HNSC, PAAD and skin cutaneous melanoma (SKCM) (Figure 5A). Furthermore, the scatter plots also illustrated that NT5E is undoubtedly highly expressed in CAFs and endothelia cells in the tumor microenvironment in above four cancers (Figures 5B–E). Moreover, the *in situ* expression of NT5E was further analyzed using HPA databases based on IHC staining, in which NT5E expression is significantly higher in tumor tissues than normal tissues, and this phenomenon is all exhibited in skin, pancreas and head and neck tissues (Figure 5F).

### 3.6 CAFs might play an important role in epithelial-mesenchymal transition of various tumor species

**FIGURE 6:**
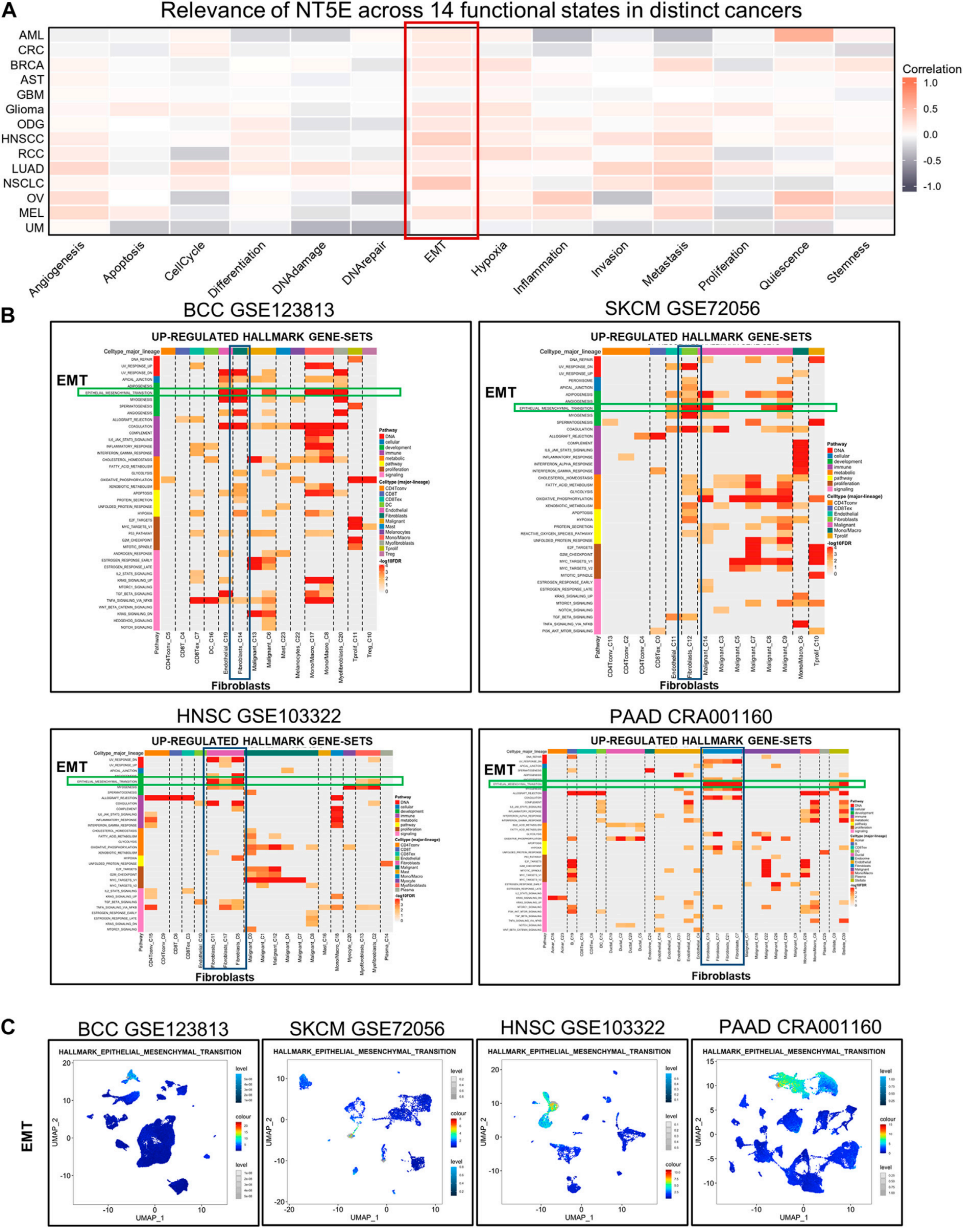
**(A)** Relevance of NT5E across 14 functional states in distinct cancers based on CancerSEA database. **(B)** The UP-REGULATED HALLMARK GENE-SETS enrichment analysis of NT5E in pan-cancer. **(C)** EMT in multiple cell subsets were obtained using the single-cell Signature Explorer function in the GSEA module of TISCH database.

Moreover, we estimated the cancer biology-related functional states of NT5E at single-cell sequencing level using CancerSEA Portal, the results exhibited that NT5E expression is positively correlated with EMT function in several cancers (Figure 6A). Then, we performed studies to obtain up-regulated Hallmark gene-sets in different cell subsets, and the results suggested that EMT function of CAFs was significantly up-regulated in multiple tumor species, including BCC, SKCM, HNSC, and PAAD (Figure 6B). Furthermore, we also found that the degree of EMT of CAFs cell subsets was higher than that of other cell subsets based on the GSEA module of TISCH database (Figure 6C). These results suggest that CAFs may play an important role in EMT of various tumor species.

### 3.7 *In-situ* immunofluorescence staining verified the NT5E expression on CAFs in HNSC specimens

**FIGURE 7:**
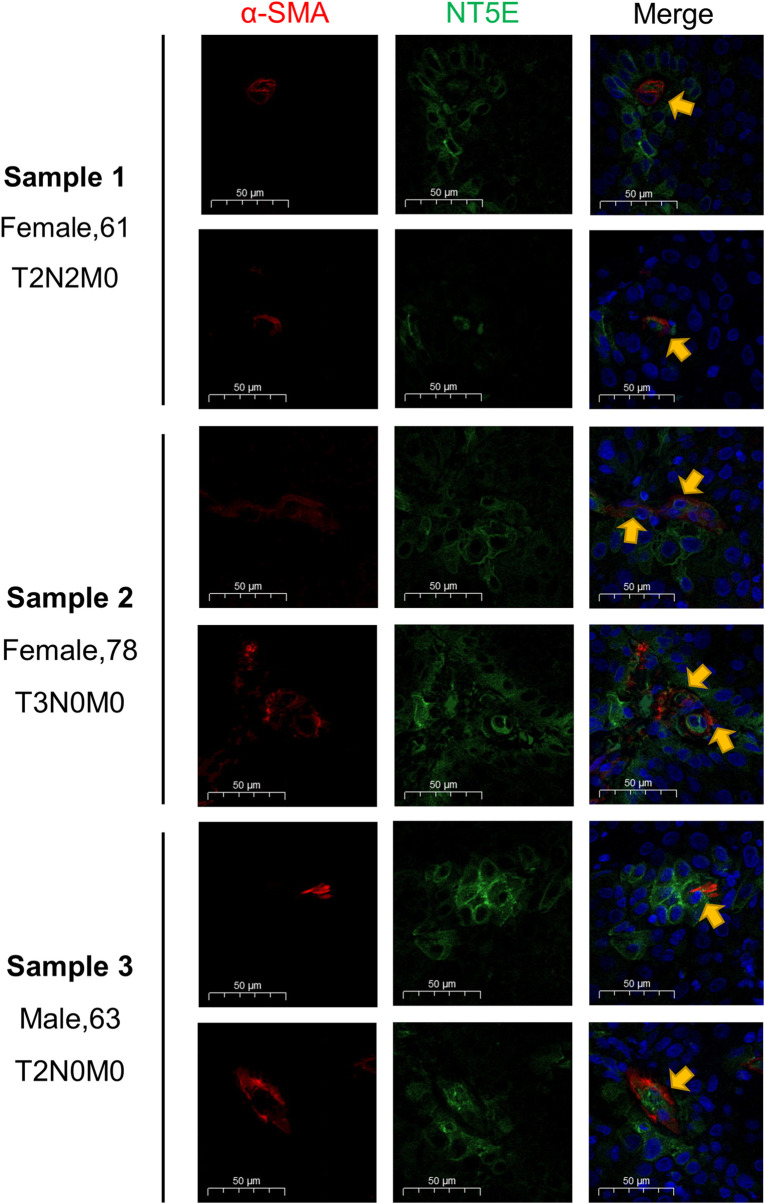
Validation of NT5E expression pattern on the supraglottic carcinoma specimens with various TNM-staging. Scale bar = 50 μm. Red stands for α-SMA expression, green stands for NT5E expression, and blue stands for nuclear staining by DAPI.

The supraglottic carcinoma specimens with various TNM-staging were collected as representatives for HNSC samples, and the staining results were displayed in Figure 7. The expression abundance of NT5E was positively related to T staging. Besides, the immunofluorescence staining results revealed the co-expressed pattern of NT5E and α-SMA, a most commonly used CAFs marker.

## 4 Discussion

This study is an integrated pan-cancer analysis about the potential prognostic value of NT5E. We reported that the expression level of NT5E was elevated in many tumor types, such as ovarian cancer and colorectal tumor tissues (Gaudreau et al., 2016; Wu et al., 2016). The overexpression of NT5E was also associated with poor prognosis, and was related to tumor development and invasion in pancreas, prostate, bladder and head and neck cancer (Yang et al., 2013; Mandapathil et al., 2018; Koivisto et al., 2019; Zhou et al., 2019; Chen et al., 2022). In this study, not only did we illustrate that NT5E might serve as a negative prognostic biomarker for LUAD, STAD, BRCA and UVM, but also revealed that it is positively correlated with tumor stage in several cancers. The results of immunofluorescence staining, in our study, also uncovered that the expression level of NT5E was positively correlated with T staging in the supraglottic carcinoma, which is considered to belong to the typical HNSC. These results suggested that NT5E might facilitate tumor growth and tumor metastasis.

Tumor metastasis is a complex process manipulated by multiple mechanisms (Pantel and Brakenhoff, 2004; Quail and Joyce, 2013; López-Soto et al., 2017). One of the important mechanisms is that the primary tumors cells invade through the physical barrier, namely the basement membrane, and disseminate *via* the circulation system. In the process of tumor invasion, the epithelial cells also break through the basement membrane and separate from neighboring cells to damage adjacent cell layers. The reason lies in the acquirement of migratory and invasive properties through EMT (Thiery et al., 2009), during which the apical-basal polarity and cell-cell adhesion of epithelial cells were weakened, and thus transited into invasive mesenchymal cells (Du and Shim, 2016). Then, mesenchymal cells could invade through ECM, one of the essential components of TME, which is composed of collagen, elastin, fibronectin, hyaluronic acid, proteoglycans and glycoproteins, undertaking the task to support tissues by encapsulating cells (Otranto et al., 2012; Pickup et al., 2014; Willumsen et al., 2018). In this case, the tumor cells would lose cell-cell adhesion and thus acquire motility. Moreover, it has been reported that the circulating tumor cells, which are important precursors of cancer metastasis, could be allowed to escape from antimetastatic checkpoints to realize distant metastasis by the mechanism of EMT process (Xiang et al., 2022). In our study, the results of enrichment analysis also demonstrated the critical role of NT5E as a regulator of cell-substrate junction, cell-substrate adherens junction, focal adhesion and external side of plasma membrane, and these functions are closely related to cell polarization and EMT (Thiery and Sleeman, 2006; Baronsky et al., 2017; Venhuizen et al., 2020), which plays an crucial role in cancer progression, especially tumor cell invasion (Son and Moon, 2010). Therefore, the pro-tumor function of NT5E may be related to facilitating the EMT of tumor cells.

Moreover, the metastatic potential of cancer cells is closely dependent on the TME. Notably, CAFs are one of the major components of the tumor stroma contributing significantly to the TME, which were differentiated from stromal fibroblast cells by the stimulation of paracrine growth factors secreted from tumor cells (Kalluri and Zeisberg, 2006; Tejada et al., 2006). Unlike normal stromal fibroblasts, CAFs could facilitate cancer cells survival (Martinez-Outschoorn et al., 2010), growth and progression (Orimo et al., 2005; Giannoni et al., 2010). It has been validated that CAFs secrete a number of cytokines, which could facilitate tumor cells invasion and metastasis by activating several signaling pathways (Ao et al., 2007; Kojima et al., 2010; Karagiannis et al., 2012; Yu et al., 2014; Shien et al., 2017; Gao et al., 2019). In addition, CAFs also play important roles in orchestrating the ECM in almost all cancers. It has been illustrated that ECM remodeling could also facilitate the invasion and migration of cancer cells, and cancer cells with the EMT phenotype make great contribution to this process by producing ECM-degrading proteases (Giannoni et al., 2010; Qiao et al., 2010). In our study, the results of enrichment analysis of NT5E may imply that it may be correlated with EMT during cancer progress. Moreover, we employed studies to gain elevated hallmark gene-sets in different cell subsets, and the results illuminated that EMT function of CAFs was elevated in BCC, SKCM, HNSC, and PAAD. Then, based on the GSEA module of TISCH database, the analysis results, also showed that the degree of EMT of CAFs was higher than that of other cell subsets. These results are consistent with the above conclusion that CAFs make great contribution to maintain tumor growth and development.

According to previous studies, a high level of CAFs infiltration was considered to make great contribute to an unfavorable clinical outcome of patients (Gieniec et al., 2019; Hosein et al., 2020; Piersma et al., 2020). Consequently, there are more and more attentions focused on the therapy targeted-CAFs, which is believed to be one of the complementary treatment strategies for cancer. The first strategy is to deplete CAFs directly by either transgenic technologies or immunotherapies (Özdemir et al., 2015). For example, the previous study revealed that a specific CAF subpopulation (referred to as CAF-S1) expressed NT5E could upregulate the power of Tregs to inhibit the proliferation of effector T cells (Costa et al., 2018; Givel et al., 2018), while this effect could be neutralize after using an anti-CD73 antibody (Magagna et al., 2021). Secondly, some molecules, including all-trans retinoic acid (ATRA) and calcipotriol, could promote CAFs to become normalized and adopt an inactive phenotype (Froeling et al., 2011). Moreover, CAFs could be used as a vehicle to deliver anticancer drugs, including TNF-related apoptosis-inducing ligand (TRAIL) or type I interferon (IFN) (Miao et al., 2017). Finally, the function of CAFs could be inhibited by regulating the activation of targeting crucial signals and effectors, such as chemokine and growth factor pathways (Giannoni et al., 2010; Albrengues et al., 2015). Interestingly, NT5E has also been considered as a novel checkpoint inhibitor target (Allard et al., 2017b). It has been illustrated that overexpression of NT5E could inhibit the immunosurveillance of immune cells, which may be correlated with immune evasion and tumor metastasis (Stagg et al., 2010; Leclerc et al., 2016). Moreover, tumor cell death could result in CAFs-NT5E overexpression *via* an adenosine-adenosinergic A2_B_ receptor (A_2B_R) mediated feedforward circuit, which could furtherly exacerbate the immunosuppressive environment (Yu et al., 2020). According to the results of our study, NT5E was related positively to the infiltration stage of CAFs in most cancers (Hu et al., 2020), and single-cell analysis also showed that NT5E was mainly expressed on the CAFs in several cancers such as BCC, HNSC, PAAD, and SKCM. Furthermore, in the supraglottic carcinoma specimens, we also illustrated that NT5E was co-expressed with α-SMA, which was one of the myofibroblast markers and expressed on CAFs (Orimo and Weinberg, 2007; Sharon et al., 2013). Thus, NT5E may be a crucial signal related to the activation of CAFs. High-NT5E expressed CAFs could serve as novel targets for immunotherapy. Thus, we performed correlation analyses between immune-related genes and NT5E expression level on pan-cancer level, and uncovered that the expression levels of several immunoinhibitors were positively correlated with NT5E expression, including kinase insert domain-containing receptor (KDR), interleukin-10 receptor B (IL10RB) and transforming growth factor-beta receptor 1 (TGFBR1) (Supplementary Figures S1, S2).

Still and all, there is no doubt that our research also has some limitations. Firstly, because the data used in this study was derived from online databases, which are characterized by open and imprecise, systematic bias may become an inevitable factor. Secondly, pan-cancer analysis is of distinct heterogeneity attributing to different cancer. Thirdly, the findings of the current investigation demand clinical trial-based validation in several cancer patients receiving high-NT5E-expression CAFs-targeting immunotherapies. Finally, more functional experiments such as flow cytometry and single cell RNA-seq are needed to further elucidate CAFs and NT5E contents in specific cancer.

## 5 Conclusion

In summary, this study is an integrated analysis of NT5E in pan-cancer. We illuminated that NT5E could serve as an efficient prognostic biomarker in pan-cancer, its expression level was positively related to CAFs and endothelial cells infiltration in pan-cancer. Moreover, NT5E is more likely expressed in the endothelia cells and CAFs in pan-cancer, and CAFs may play an important role in EMT of various tumor species. We concluded that the underlying mechanism of the NT5E pro-tumor effect may be related to the up-regulated EMT function of CAFs, which may provide some information to study immune therapy targeted CAFs and NT5E.

## Data Availability

The original contributions presented in the study are included in the article/Supplementary Materials, further inquiries can be directed to the corresponding author/s.

## References

[B1] Alam M. S. Kurtz C. C. Rowlett R. M. Reuter B. K. Wiznerowicz E. Das S. (2009). CD73 is expressed by human regulatory T helper cells and suppresses proinflammatory cytokine production and Helicobacter felis-induced gastritis in mice. J. Infect. Dis. 199 (4), 494–504. 10.1086/596205 19281303 PMC3047419

[B2] Albrengues J. Bertero T. Grasset E. Bonan S. Maiel M. Bourget I. (2015). Epigenetic switch drives the conversion of fibroblasts into proinvasive cancer-associated fibroblasts. Nat. Commun. 6, 10204. 10.1038/Ncomms10204 26667266 PMC4682161

[B3] Allard B. Beavis P. A. Darcy P. K. Stagg J. (2016). Immunosuppressive activities of adenosine in cancer. Curr. Opin. Pharmacol. 29, 7–16. 10.1016/J.Coph.2016.04.001 27209048

[B4] Allard B. Longhi M. S. Robson S. C. Stagg J. (2017). The ectonucleotidases CD39 and CD73: Novel checkpoint inhibitor targets. Immunol. Rev. 276 (1), 121–144. 10.1111/Imr.12528 28258700 PMC5338647

[B5] Allard B. Turcotte M. Stagg J. (2014). Targeting CD73 and downstream adenosine receptor signaling in triple-negative breast cancer. Expert Opin. Ther. Targets 18 (8), 863–881. 10.1517/14728222.2014.915315 24798880

[B6] Allard D. Turcotte M. Stagg J. (2017). Targeting A2 adenosine receptors in cancer. Immunol. Cell Biol. 95 (4), 333–339. 10.1038/Icb.2017.8 28174424

[B7] Antonioli L. Blandizzi C. Pacher P. Hasko G. (2013). Immunity, inflammation and cancer: a leading role for adenosine. Nat. Rev. Cancer 13 (12), 842–857. 10.1038/Nrc3613 24226193

[B8] Ao M. Franco O. E. Park D. Raman D. Williams K. Hayward S. W. (2007). Cross-talk between paracrine-acting cytokine and chemokine pathways promotes malignancy in benign human prostatic epithelium. Cancer Res. 67 (9), 4244–4253. 10.1158/0008-5472.Can-06-3946 17483336

[B9] Baronsky T. Ruhlandt D. Brückner B. R. Schafer J. Karedla N. Isbaner S. (2017). Cell-substrate dynamics of the epithelial-to-mesenchymal transition. Nano Lett. 17 (5), 3320–3326. 10.1021/Acs.Nanolett.7b01558 28440076

[B10] Berchtold S. Ogilvie A. L. Bogdan C. Muhl-Zurbes P. Schuler G. (1999). Human monocyte derived dendritic cells express functional P2X and P2Y receptors as well as ecto-nucleotidases. FEBS Lett. 458 (3), 424–428. 10.1016/S0014-5793(99)01197-7 10570953

[B11] Binnewies M. Roberts E. W. Kersten K. Chan V. Fearon D. F. Merad M. (2018). Understanding the tumor immune microenvironment (TIME) for effective therapy. Nat. Med. 24 (5), 541–550. 10.1038/S41591-018-0014-X 29686425 PMC5998822

[B12] Boison D. Yegutkin G. G. (2019). Adenosine metabolism: Emerging concepts for cancer therapy. Cancer Cell 36 (6), 582–596. 10.1016/J.Ccell.2019.10.007 31821783 PMC7224341

[B13] Bouma M. G. Van Den Wildenberg F. A. Buurman W. A. (1996). Adenosine inhibits cytokine release and expression of adhesion molecules by activated human endothelial cells. Am. J. Physiol. 270, C522–C529. 10.1152/Ajpcell.1996.270.2.C522 8779915

[B14] Buisseret L. Pommey S. Allard B. Garaud S. Bergeron M. Cousineau I. (2018). Clinical significance of CD73 in triple-negative breast cancer: multiplex analysis of a phase III clinical trial. Ann. Oncol. 29 (4), 1056–1062. 10.1093/Annonc/Mdx730 29145561 PMC5913595

[B15] Buschette-Brambrink S. Gutensohn W. (1989). Human placental ecto-5'-nucleotidase: Isoforms and chemical crosslinking products of the membrane-bound and isolated enzyme. Biol. Chem. Hoppe. Seyler. 370 (1), 67–74. 10.1515/Bchm3.1989.370.1.67 2540766

[B16] Chen X. M. Liu Y. Y. Tao B. Y. Xue X. M. Zhang X. X. Wang L. L. (2022). NT5E upregulation in head and neck squamous cell carcinoma: a novel biomarker on cancer-associated fibroblasts for predicting immunosuppressive tumor microenvironment. Front. Immunol. 13, 97. 10.3389/Fimmu.2022.975847 PMC9458906 36091055

[B17] Cirri P. Chiarugi P. (2011). Cancer associated fibroblasts: the dark side of the coin. Am. J. Cancer Res. 1 (4), 482–497. 21984967 PMC3186047

[B18] Colella M. Zinni M. Pansiot J. Cassanello M. Mairesse J. Ramenghi L. (2018). Modulation of microglial activation by adenosine A2a receptor in animal models of perinatal brain injury. Front. Neurol. 9, 605. 10.3389/Fneur.2018.00605 30254599 PMC6141747

[B19] Costa A. Kieffer Y. Scholer-Dahirel A. Pelon F. Bourachot B. Cardon M. (2018). Fibroblast heterogeneity and immunosuppressive environment in human breast cancer. Cancer Cell 33 (3), 463–479. 10.1016/J.Ccell.2018.01.011 29455927

[B20] Costa A. Scholer-Dahirel A. Mechta-Grigoriou F. (2014). The role of reactive oxygen species and metabolism on cancer cells and their microenvironment. Semin. Cancer Biol. 25, 23–32. 10.1016/J.Semcancer.2013.12.007 24406211

[B21] Du B. Shim J. S. (2016). Targeting epithelial-mesenchymal transition (EMT) to overcome drug resistance in cancer. Molecules 21 (7), 965. 10.3390/Molecules21070965 27455225 PMC6273543

[B22] Fini C. Talamo F. Cherri S. Coli M. Floridi A. Ferrara L. (2003). Biochemical and mass spectrometric characterization of soluble ecto-5'-nucleotidase from bull seminal plasma. Biochem. J. 372 (2), 443–451. 10.1042/Bj20021687 12608891 PMC1223402

[B23] Froeling F. E. Feig C. Chelala C. Dobson R. Mein C. E. Tuveson D. A. (2011). Retinoic acid-induced pancreatic stellate cell quiescence reduces paracrine Wnt-β-catenin signaling to slow tumor progression. Gastroenterology 141 (4), 1486–1497. 10.1053/J.Gastro.2011.06.047 21704588

[B24] Gajewski T. F. (2015). The next hurdle in cancer immunotherapy: Overcoming the non-T-cell-inflamed tumor microenvironment. Semin. Oncol. 42 (4), 663–671. 10.1053/J.Seminoncol.2015.05.011 26320069 PMC4555998

[B25] Gao Q. Yang Z. Xu S. Li X. Yang X. Jin P. (2019). Heterotypic CAF-tumor spheroids promote early peritoneal metastatis of ovarian cancer. J. Exp. Med. 216 (3), 688–703. 10.1084/Jem.20180765 30710055 PMC6400537

[B26] Gascard P. Tlsty T. D. (2016). Carcinoma-associated fibroblasts: orchestrating the composition of malignancy. Genes Dev. 30 (9), 1002–1019. 10.1101/Gad.279737.116 27151975 PMC4863733

[B27] Gaudreau P. O. Allard B. Turcotte M. Stagg J. (2016). CD73-adenosine reduces immune responses and survival in ovarian cancer patients. Oncoimmunology 5 (5), E1127496. 10.1080/2162402x.2015.1127496 27467942 PMC4910753

[B28] Gentric G. Mieulet V. Mechta-Grigoriou F. (2017). Heterogeneity in cancer metabolism: New concepts in an old field. Antioxid. Redox Signal. 26 (9), 462–485. 10.1089/Ars.2016.6750 27228792 PMC5359687

[B29] Giannoni E. Bianchini F. Masieri L. Serni S. Torre E. Calorini L. (2010). Reciprocal activation of prostate cancer cells and cancer-associated fibroblasts stimulates epithelial-mesenchymal transition and cancer stemness. Cancer Res. 70 (17), 6945–6956. 10.1158/0008-5472.Can-10-0785 20699369

[B30] Gieniec K. A. Butler L. M. Worthley D. L. Woods S. L. (2019). Cancer-associated fibroblasts-heroes or villains? Br. J. Cancer 121 (4), 293–302. 10.1038/S41416-019-0509-3 31289350 PMC6738083

[B31] Givel A. M. Kieffer Y. Scholer-Dahirel A. Sirven P. Cardon M. Pelon F. (2018). miR200-regulated CXCL12β promotes fibroblast heterogeneity and immunosuppression in ovarian cancers. Nat. Commun. 9 (1), 1056. 10.1038/S41467-018-03348-Z 29535360 PMC5849633

[B32] Goulioumis A. Gyftopoulos K. (2022). Epithelial-to-Mesenchymal transition in metastasis: Focus on laryngeal carcinoma. Biomedicines 10 (9), 2148. 10.3390/Biomedicines10092148 36140250 PMC9496235

[B33] Henttinen T. Jalkanen S. Yegutkin G. G. (2003). Adherent leukocytes prevent adenosine formation and impair endothelial barrier function by Ecto-5'-nucleotidase/CD73-dependent mechanism. J. Biol. Chem. 278 (27), 24888–24895. 10.1074/Jbc.M300779200 12707258

[B34] Hosein A. N. Brekken R. A. Maitra A. (2020). Pancreatic cancer stroma: an update on therapeutic targeting strategies. Nat. Rev. Gastroenterol. Hepatol. 17 (8), 487–505. 10.1038/S41575-020-0300-1 32393771 PMC8284850

[B35] Hu G. Cheng P. Pan J. Wang S. Ding Q. Jiang Z. (2020). An IL6-adenosine positive feedback loop between CD73+ γδTregs and CAFs promotes tumor progression in human breast cancer. Cancer Immunol. Res. 8 (10), 1273–1286. 10.1158/2326-6066.Cir-19-0923 32847938

[B36] Inoue Y. Yoshimura K. Kurabe N. Kahyo T. Kawase A. Tanahashi M. (2017). Prognostic impact of CD73 and A2A adenosine receptor expression in non-small-cell lung cancer. Oncotarget 8 (5), 8738–8751. 10.18632/Oncotarget.14434 28060732 PMC5352437

[B37] Jacob M. Chang L. Puré E. (2012). Fibroblast activation protein in remodeling tissues. Curr. Mol. Med. 12 (10), 1220–1243. 10.2174/156652412803833607 22834826

[B38] Jeong Y. J. Oh H. K. Choi H. R. (2020). Methylation Of The Nt5e Gene Is Associated With Poor Prognostic Factors In Breast Cancer. Diagn. (Basel) 10 (11). 10.3390/Diagnostics10110939 PMC7697174 33198064

[B39] Kalekar L. A. Mueller D. L. (2017). Relationship between CD4 regulatory T cells and anergy *in vivo* . J. Immunol. 198 (7), 2527–2533. 10.4049/Jimmunol.1602031 28320913 PMC5363282

[B40] Kalekar L. A. Schmiel S. E. Nandiwada S. L. Lam W. Y. Barsness L. O. Zhang N. (2016). CD4(+) T cell anergy prevents autoimmunity and generates regulatory T cell precursors. Nat. Immunol. 17 (3), 304–314. 10.1038/Ni.3331 26829766 PMC4755884

[B41] Kalluri R. (2016). The biology and function of fibroblasts in cancer. Nat. Rev. Cancer 16 (9), 582–598. 10.1038/Nrc.2016.73 27550820

[B42] Kalluri R. Zeisberg M. (2006). Fibroblasts in cancer. Nat. Rev. Cancer 6 (5), 392–401. 10.1038/Nrc1877 16572188

[B43] Karagiannis G. S. Poutahidis T. Erdman S. E. Kirsch R. Riddell R. H. Diamandis E. P. (2012). Cancer-associated fibroblasts drive the progression of metastasis through both paracrine and mechanical pressure on cancer tissue. Mol. Cancer Res. 10 (11), 1403–1418. 10.1158/1541-7786.Mcr-12-0307 23024188 PMC4399759

[B44] Koivisto M. K. Tervahartiala M. Kenessey I. Jalkanen S. Bostrom P. J. Salmi M. (2019). Cell-type-specific CD73 expression is an independent prognostic factor in bladder cancer. Carcinogenesis 40 (1), 84–92. 10.1093/Carcin/Bgy154 30395172

[B45] Kojima Y. Acar A. Eaton E. N. Mellody K. T. Scheel C. Ben-Porath I. (2010). Autocrine TGF-beta and stromal cell-derived factor-1 (SDF-1) signaling drives the evolution of tumor-promoting mammary stromal myofibroblasts. Proc. Natl. Acad. Sci. U. S. A. 107 (46), 20009–20014. 10.1073/Pnas.1013805107 21041659 PMC2993333

[B46] Kordas T. Osen W. Eichmüller S. B. (2018). Controlling the immune suppressor: Transcription factors and MicroRNAs regulating CD73/NT5E. Front. Immunol. 9, 813. 10.3389/Fimmu.2018.00813 29720980 PMC5915482

[B47] Leclerc B. G. Charlebois R. Chouinard G. Allard B. Pommey S. Saad F. (2016). CD73 expression is an independent prognostic factor in prostate cancer. Clin. Cancer Res. 22 (1), 158–166. 10.1158/1078-0432.Ccr-15-1181 26253870

[B48] Leone R. D. Emens L. A. (2018). Targeting adenosine for cancer immunotherapy. J. Immunother. Cancer 6 (1), 57. 10.1186/S40425-018-0360-8 29914571 PMC6006764

[B49] Liu N. Fang X. D. Vadis Q. (2012). CD73 as a novel prognostic biomarker for human colorectal cancer. J. Surg. Oncol. 106 (7), 918–919. 10.1002/Jso.23159 22585744

[B50] López-Soto A. Gonzalez S. Smyth M. J. Galluzzi L. (2017). Control of metastasis by NK cells. Cancer Cell 32 (2), 135–154. 10.1016/J.Ccell.2017.06.009 28810142

[B51] Magagna I. Gourdin N. Kieffer Y. Licaj M. Mhaidly R. Andre P. (2021). CD73-Mediated immunosuppression is linked to a specific fibroblast population that paves the way for new therapy in breast cancer. Cancers (Basel) 13 (23), 5878. 10.3390/Cancers13235878 34884993 PMC8657241

[B52] Mandapathil M. Boduc M. Netzer C. Guldner C. Roessler M. Wallicek-Dworschak U. (2018). CD73 expression in lymph node metastases in patients with head and neck cancer. Acta Otolaryngol. 138 (2), 180–184. 10.1080/00016489.2017.1378436 28938850

[B53] Marsh T. Pietras K. Mcallister S. S. (2013). Fibroblasts as architects of cancer pathogenesis. Biochim. Biophys. Acta 1832 (7), 1070–1078. 10.1016/J.Bbadis.2012.10.013 23123598 PMC3775582

[B54] Martinez-Outschoorn U. E. Trimmer C. Lin Z. Whitaker-Menezes D. Chiavarina B. Zhou J. (2010). Autophagy in cancer associated fibroblasts promotes tumor cell survival: Role of hypoxia, HIF1 induction and NFκB activation in the tumor stromal microenvironment. Cell Cycle 9 (17), 3515–3533. 10.4161/Cc.9.17.12928 20855962 PMC3047617

[B55] Miao L. Liu Q. Lin C. M. Luo C. Wang Y. (2017). Targeting tumor-associated fibroblasts for therapeutic delivery in desmoplastic tumors. Cancer Res. 77 (3), 719–731. 10.1158/0008-5472.Can-16-0866 27864344 PMC5290135

[B56] Neo S. Y. Yang Y. Record J. Ma R. Chen X. Chen Z. (2020). CD73 immune checkpoint defines regulatory NK cells within the tumor microenvironment. J. Clin. Invest. 130 (3), 1185–1198. 10.1172/Jci128895 31770109 PMC7269592

[B57] Obayashi T. Kagaya Y. Aoki Y. Tadaka S. Kinoshita K. (2019). COXPRESdb v7: a gene coexpression database for 11 animal species supported by 23 coexpression platforms for technical evaluation and evolutionary inference. Nucleic Acids Res. 47 (D1), D55–D62. 10.1093/Nar/Gky1155 30462320 PMC6324053

[B58] Orimo A. Gupta P. B. Sgroi D. C. Arenzana-Seisdedos F. Delaunay T. Naeem R. (2005). Stromal fibroblasts present in invasive human breast carcinomas promote tumor growth and angiogenesis through elevated SDF-1/CXCL12 secretion. Cell 121 (3), 335–348. 10.1016/J.Cell.2005.02.034 15882617

[B59] Orimo A. Weinberg R. A. (2007). Heterogeneity of stromal fibroblasts in tumors. Cancer Biol. Ther. 6 (4), 618–619. 10.4161/Cbt.6.4.4255 18027438

[B60] Otranto M. Sarrazy V. Bonté F. Hinz B. Gabbiani G. Desmouliere A. (2012). The role of the myofibroblast in tumor stroma remodeling. Cell adh. Migr. 6 (3), 203–219. 10.4161/Cam.20377 22568985 PMC3427235

[B61] Özdemir B. C. Pentcheva-Hoang T. Carstens J. L. Zheng X. Wu C. C. Simpson T. R. (2015). Depletion of carcinoma-associated fibroblasts and fibrosis induces immunosuppression and accelerates pancreas cancer with reduced survival. Cancer Cell 28 (6), 831–833. 10.1016/J.Ccell.2015.11.002 28843279

[B62] Pantel K. Brakenhoff R. H. (2004). Dissecting the metastatic cascade. Nat. Rev. Cancer 4 (6), 448–456. 10.1038/Nrc1370 15170447

[B63] Pellegatti P. Raffaghello L. Bianchi G. Piccardi F. Pistoia V. Di Virgilio F. (2008). Increased level of extracellular ATP at tumor sites: *in vivo* imaging with plasma membrane luciferase. Plos One 3 (7), E2599. 10.1371/Journal.Pone.0002599 18612415 PMC2440522

[B64] Pickup M. W. Mouw J. K. Weaver V. M. (2014). The extracellular matrix modulates the hallmarks of cancer. EMBO Rep. 15 (12), 1243–1253. 10.15252/Embr.201439246 25381661 PMC4264927

[B65] Piersma B. Hayward M. K. Weaver V. M. (2020). Fibrosis and cancer: A strained relationship. Biochim. Biophys. Acta. Rev. Cancer 1873 (2), 188356. 10.1016/J.Bbcan.2020.188356 32147542 PMC7733542

[B66] Qiao B. Johnson N. W. Gao J. (2010). Epithelial-mesenchymal transition in oral squamous cell carcinoma triggered by transforming growth factor-beta1 is Snail family-dependent and correlates with matrix metalloproteinase-2 and -9 expressions. Int. J. Oncol. 37 (3), 663–668. 10.3892/Ijo_00000715 20664935

[B67] Quail D. F. Joyce J. A. (2013). Microenvironmental regulation of tumor progression and metastasis. Nat. Med. 19 (11), 1423–1437. 10.1038/Nm.3394 24202395 PMC3954707

[B68] Quail D. F. Joyce J. A. (2017). The microenvironmental landscape of brain tumors. Cancer Cell 31 (3), 326–341. 10.1016/J.Ccell.2017.02.009 28292436 PMC5424263

[B69] Ryzhov S. Novitskiy S. V. Goldstein A. E. Biktasova A. Blackburn M. R. Biaggioni I. (2011). Adenosinergic regulation of the expansion and immunosuppressive activity of CD11b+Gr1+ cells. J. Immunol. 187 (11), 6120–6129. 10.4049/Jimmunol.1101225 22039302 PMC3221925

[B70] Sadej R. Skladanowski A. C. (2012). Dual, enzymatic and non-enzymatic, function of ecto-5'-nucleotidase (eN, CD73) in migration and invasion of A375 melanoma cells. Acta Biochim. Pol. 59 (4), 647–652. 10.18388/abp.2012_2105 23162807

[B71] Sadej R. Spychala J. Skladanowski A. C. (2006). Ecto-5'-nucleotidase (eN, CD73) is coexpressed with metastasis promoting antigens in human melanoma cells. Nucleosides Nucleotides Nucleic Acids 25 (9-11), 1119–1123. 10.1080/15257770600894188 17065075

[B72] Sadej R. Spychala J. Skladanowski A. C. (2006). Expression of ecto-5'-nucleotidase (eN, CD73) in cell lines from various stages of human melanoma. Melanoma Res. 16 (3), 213–222. 10.1097/01.Cmr.0000215030.69823.11 16718268

[B73] Saldanha-Araujo F. Ferreira F. I. Palma P. V. Araujo A. G. Queiroz R. H. C. Covas D. T. (2011). Mesenchymal stromal cells up-regulate CD39 and increase adenosine production to suppress activated T-lymphocytes. Stem Cell Res. 7 (1), 66–74. 10.1016/J.Scr.2011.04.001 21546330

[B74] Sharon Y. Alon L. Glanz S. Servais C. Erez N. (2013). Isolation of normal and cancer-associated fibroblasts from fresh tissues by Fluorescence Activated Cell Sorting (FACS). J. Vis. Exp. (71), e4425. 10.3791/4425 23354290 PMC3582516

[B75] Shien K. Papadimitrakopoulou V. A. Ruder D. Behrens C. Shen L. Kalhor N. (2017). JAK1/STAT3 activation through a proinflammatory cytokine pathway leads to resistance to molecularly targeted therapy in non-small cell lung cancer. Mol. Cancer Ther. 16 (10), 2234–2245. 10.1158/1535-7163.Mct-17-0148 28729401 PMC5628136

[B76] Sitkovsky M. V. Lukashev D. Apasov S. Kojima H. Koshiba M. Caldwell C. (2004). Physiological control of immune response and inflammatory tissue damage by hypoxia-inducible factors and adenosine A2A receptors. Annu. Rev. Immunol. 22, 657–682. 10.1146/Annurev.Immunol.22.012703.104731 15032592

[B77] Son H. Moon A. (2010). Epithelial-mesenchymal transition and cell invasion. Toxicol. Res. 26 (4), 245–252. 10.5487/Tr.2010.26.4.245 24278531 PMC3834497

[B78] Spychala J. (2000). Tumor-promoting functions of adenosine. Pharmacol. Ther. 87 (2-3), 161–173. 10.1016/S0163-7258(00)00053-X 11007998

[B79] Stagg J. Divisekera U. Mclaughlin N. Sharkey J. Pommey S. Denoyer D. (2010). Anti-CD73 antibody therapy inhibits breast tumor growth and metastasis. Proc. Natl. Acad. Sci. U. S. A. 107 (4), 1547–1552. 10.1073/Pnas.0908801107 20080644 PMC2824381

[B80] Sträter N. (2006). Ecto-5'-nucleotidase: structure function relationships. Purinergic Signal. 2 (2), 343–350. 10.1007/S11302-006-9000-8 18404474 PMC2254484

[B81] Tejada M. L. Yu L. Dong J. Jung K. Meng G. Peale F. V. (2006). Tumor-driven paracrine platelet-derived growth factor receptor alpha signaling is a key determinant of stromal cell recruitment in a model of human lung carcinoma. Clin. Cancer Res. 12 (9), 2676–2688. 10.1158/1078-0432.Ccr-05-1770 16675559

[B82] Thiery J. P. Acloque H. Huang R. Y. Nieto M. A. (2009). Epithelial-mesenchymal transitions in development and disease. Cell 139 (5), 871–890. 10.1016/J.Cell.2009.11.007 19945376

[B83] Thiery J. P. Sleeman J. P. (2006). Complex networks orchestrate epithelial-mesenchymal transitions. Nat. Rev. Mol. Cell Biol. 7 (2), 131–142. 10.1038/Nrm1835 16493418

[B84] Thompson L. F. Eltzschig H. K. Ibla J. C. Van De Wiele C. J. Resta R. Morote-Garcia J. C. (2004). Crucial role for ecto-5'-nucleotidase (CD73) in vascular leakage during hypoxia. J. Exp. Med. 200 (11), 1395–1405. 10.1084/Jem.20040915 15583013 PMC1237012

[B85] Venhuizen J. H. Jacobs F. J. C. Span P. N. Zegers M. M. (2020). P120 and E-cadherin: Double-edged swords in tumor metastasis. Semin. Cancer Biol. 60, 107–120. 10.1016/J.Semcancer.2019.07.020 31369816

[B86] Wang H. Lee S. Nigro C. L. Lattanzio L. Merlano M. Monteverde M. (2012). NT5E (CD73) is epigenetically regulated in malignant melanoma and associated with metastatic site specificity. Br. J. Cancer 106 (8), 1446–1452. 10.1038/Bjc.2012.95 22454080 PMC3326678

[B87] Wei C. Ma Y. Wang F. Liao Y. Chen Y. Zhao B. (2022). Igniting hope for tumor immunotherapy: Promoting the "hot and cold" tumor transition. Clin. Med. Insights. Oncol. 16, 11795549221120708. 10.1177/11795549221120708 36147198 PMC9486259

[B88] Willumsen N. Thomsen L. B. Bager C. L. Jensen C. Karsdal M. A. (2018). Quantification of altered tissue turnover in a liquid biopsy: a proposed precision medicine tool to assess chronic inflammation and desmoplasia associated with a pro-cancerous niche and response to immuno-therapeutic anti-tumor modalities. Cancer Immunol. Immunother. 67 (1), 1–12. 10.1007/S00262-017-2074-Z 29022089 PMC11028250

[B89] Wu R. Chen Y. Li F. Li W. Zhou H. Yang Y. (2016). Effects of CD73 on human colorectal cancer cell growth *in vivo* and *in vitro* . Oncol. Rep. 35 (3), 1750–1756. 10.3892/Or.2015.4512 26708311

[B90] Xiang Z. Huang G. Wu H. He Q. Yang C. Dou R. (2022). SNHG16 upregulation-induced positive feedback loop with YAP1/TEAD1 complex in Colorectal Cancer cell lines facilitates liver metastasis of colorectal cancer by modulating CTCs epithelial-mesenchymal transition. Int. J. Biol. Sci. 18 (14), 5291–5308. 10.7150/Ijbs.73438 36147462 PMC9461660

[B91] Xu Z. Gu C. Yao X. Guo W. Wang H. Lin T. (2020). CD73 promotes tumor metastasis by modulating RICS/RhoA signaling and EMT in gastric cancer. Cell Death Dis. 11 (3), 202. 10.1038/S41419-020-2403-6 32205841 PMC7089986

[B92] Yang Q. Du J. Zu L. (2013). Overexpression of CD73 in prostate cancer is associated with lymph node metastasis. Pathol. Oncol. Res. 19 (4), 811–814. 10.1007/S12253-013-9648-7 23653114

[B93] Young A. Ngiow S. F. Barkauskas D. S. Sult E. Hay C. Blake S. J. (2016). Co-Inhibition of CD73 and A2AR adenosine signaling improves anti-tumor immune responses. Cancer Cell 30 (3), 391–403. 10.1016/J.Ccell.2016.06.025 27622332

[B94] Yu M. Guo G. Huang L. Deng L. Chang C. S. Achyut B. R. (2020). CD73 on cancer-associated fibroblasts enhanced by the A2B-mediated feedforward circuit enforces an immune checkpoint. Nat. Commun. 11 (1), 515. 10.1038/S41467-019-14060-X 31980601 PMC6981126

[B95] Yu Y. Xiao C. H. Tan L. D. Wang Q. S. Li X. Q. Feng Y. M. (2014). Cancer-associated fibroblasts induce epithelial-mesenchymal transition of breast cancer cells through paracrine TGF-β signalling. Br. J. Cancer 110 (3), 724–732. 10.1038/Bjc.2013.768 24335925 PMC3915130

[B96] Zhou L. Jia S. Chen Y. Wang W. Wu Z. Yu W. (2019). The distinct role of CD73 in the progression of pancreatic cancer. J. Mol. Med. 97 (6), 803–815. 10.1007/S00109-018-01742-0 30927045 PMC6525710

[B97] Zhu X. Liang R. Lan T. Ding D. Huang S. Shao J. (2022). Tumor-associated macrophage-specific CD155 contributes to M2-phenotype transition, immunosuppression, and tumor progression in colorectal cancer. J. Immunother. Cancer 10 (9), e004219. 10.1136/Jitc-2021-004219 36104099 PMC9476138

[B98] Zimmermann H. Zebisch M. Sträter N. (2012). Cellular function and molecular structure of ecto-nucleotidases. Purinergic Signal 8 (3), 437–502. 10.1007/S11302-012-9309-4 22555564 PMC3360096

[B99] Zimmermann H. (2000). Extracellular metabolism of ATP and other nucleotides. Naunyn. Schmiedeb. Arch. Pharmacol. 362 (4-5), 299–309. 10.1007/S002100000309 11111825

