# Supplementary figures and images for: Pan-cancer analysis identifies NT5E as a novel prognostic biomarker on cancer-associated fibroblasts associated with unique tumor microenvironment
Source: Front Pharmacol. 2022 Dec 7;13:1064032. doi: 10.3389/fphar.2022.1064032 (PMC9768042; doi:10.3389/fphar.2022.1064032)

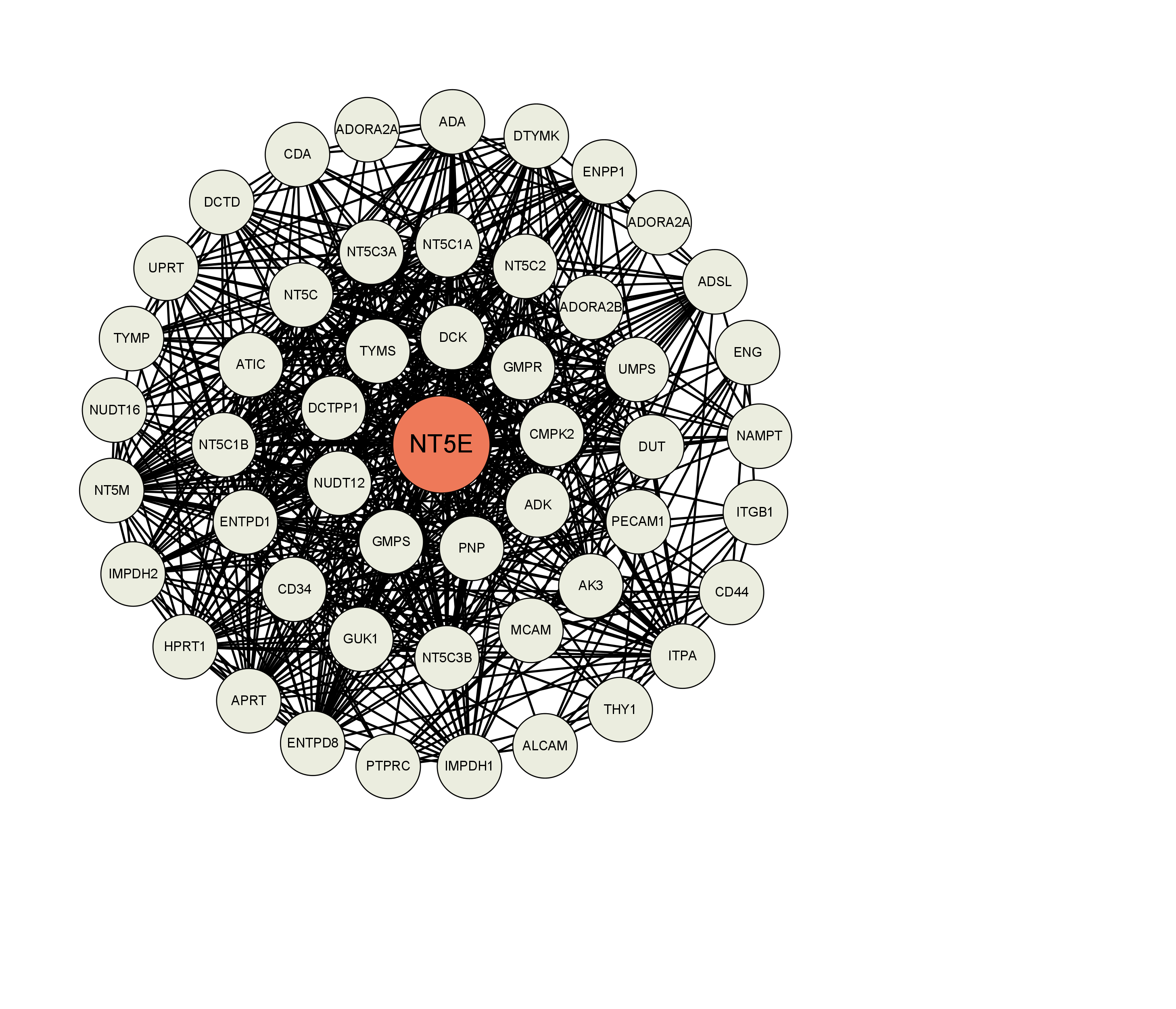

Supplement: Supplementary file 1 [file DataSheet1.ZIP › raw data/Figure4-A-string_interactions_short.tsv.png]

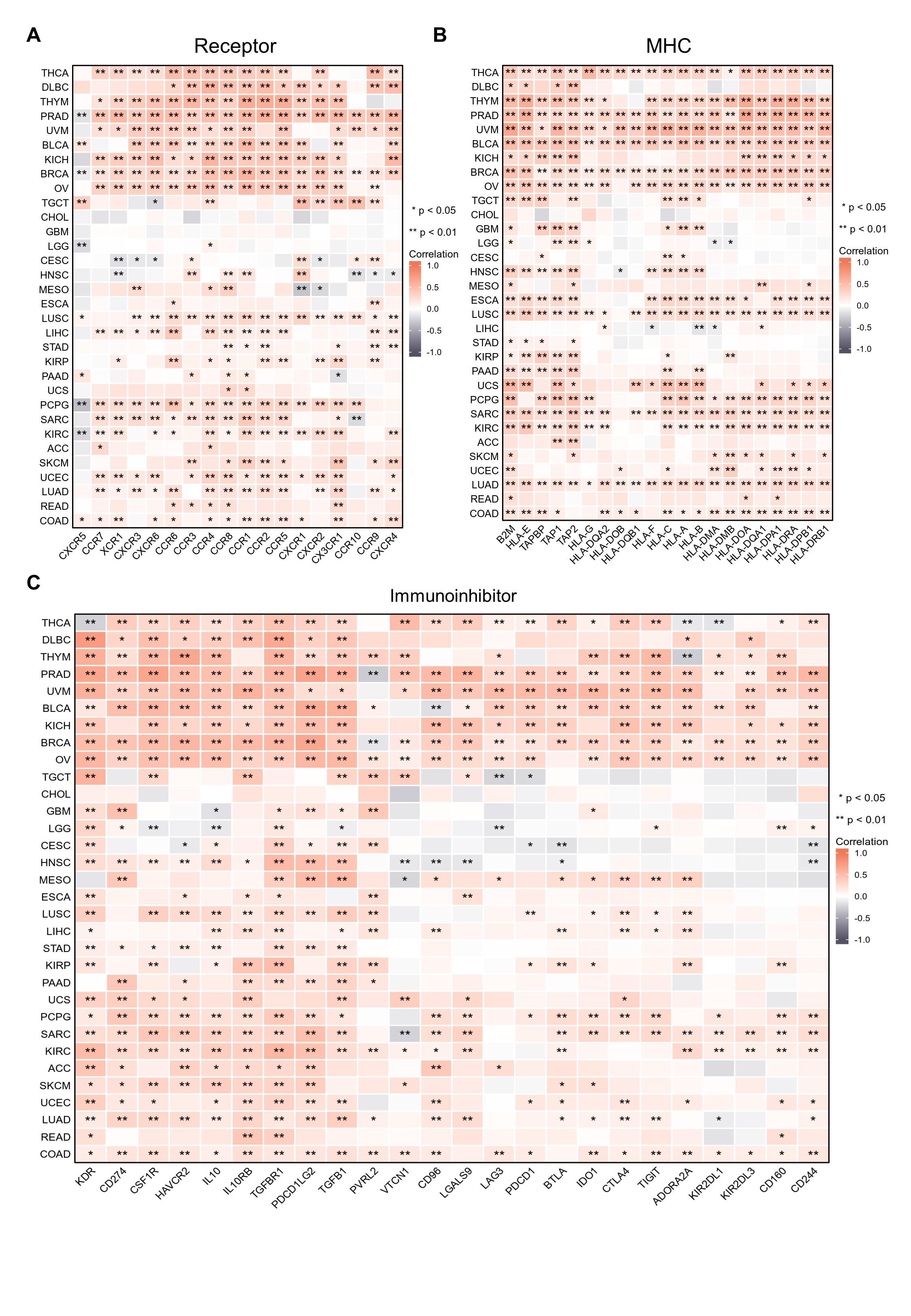

Supplement: Supplementary file 2 [file Image2.TIF]

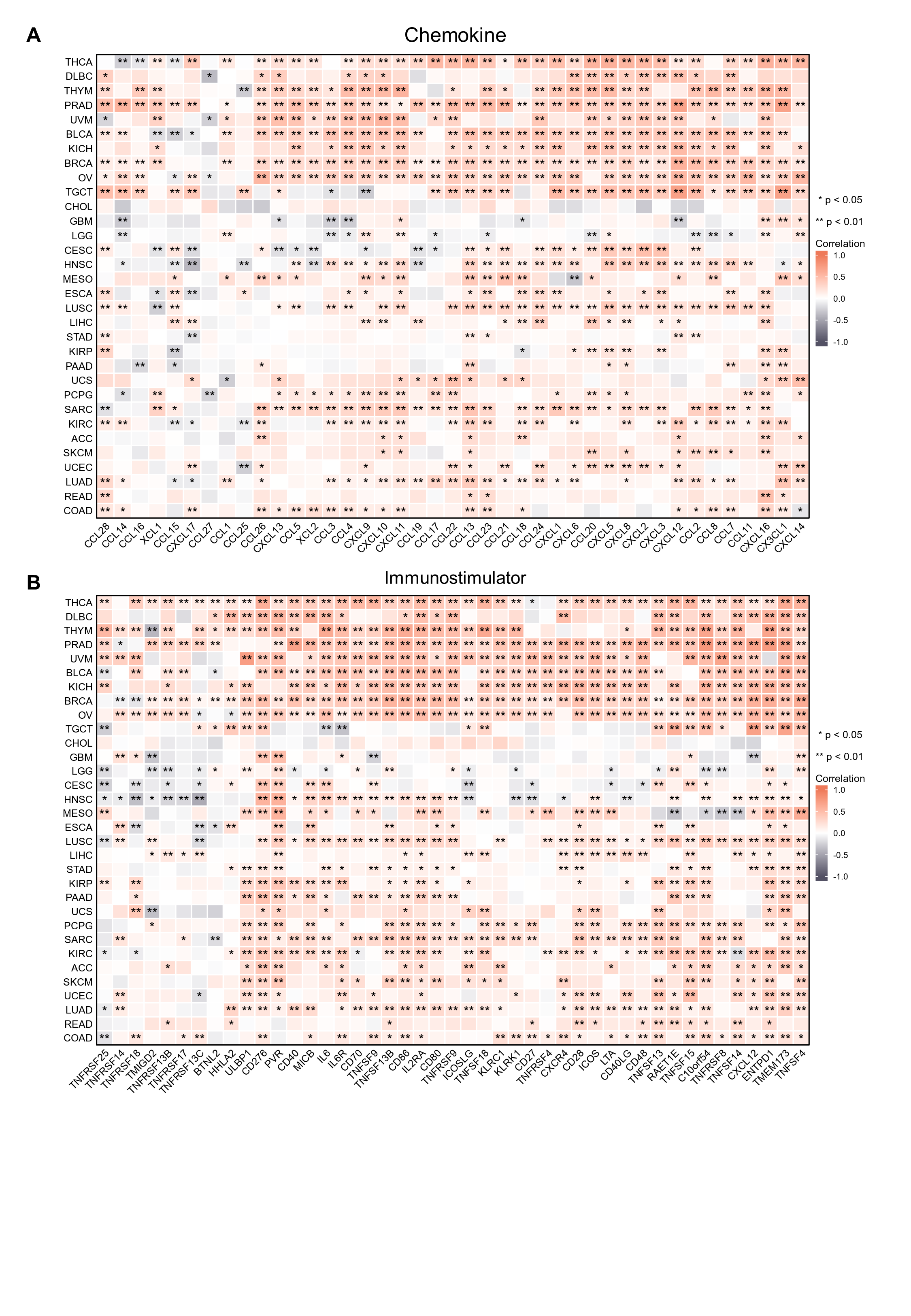

Supplement: Supplementary file 3 [file Image1.TIF]
